# Four Letters with Answers

**Published:** 1881-07

**Authors:** 


### Our Correspondence.

BATH, N. Y., April 1.

In your article on Electric Brushes you attack "Edison" for lending his name to an imposition. The "Electric Garter" is not *Edison's* but *Edson's*—of course the intent of the makers is to deceive by giving a name very similar to the great inventor's. The Electric Garter Co. I refer to is of London, England. Have you not done Mr. E. an injustice ?

A BATH READER.

Perhaps we were in error in reading the name; but, if Mr. Edison lends his name to a patent medicine swindle—like his "polyform," he is not above the electric brush or garter humbug.

---

BUFFALO, Mich. 31, 1881.

DEAR DOCTOR:—In your article on "Glass Drilling," you say prepare camphor and turpentine, etc., and make the drill size of hole required. I simply use turpentine and a three-sided common file with *not* a too fine point, and can drill a hole very easy any size necessary. I do this frequently, and sometimes in very thin glass vessels.

Put me down in your list as a two years' subscriber. I see there is a chance not only for instruction, but amusement also, in "BISTOURY.' Yours, HARTZ.

As Prof. Hartz is up to tricks of this kind, his experience is worth recording.

---

Now, comes a chap who relates his experience with felons. He seems to have poulticed it for a week, and sweat it for two weeks more, and then credits the bath with the cure. It should have "died a natural death" in that while. But, since he derived comfort and consolation from his "sweat," and was led to *believe* that it cured him and his felon, that is all any reasonable man can demand of any cure:

NEW YORK, June 7, 1881.

DEAR DOCTOR:

Seeing in your BISTOURY of April an article headed, "Domestic remedy for felon," I will give you my experience, which is a novel one. I was taken with the feeling that a sliver had been deposited under the skin; it became sore and I cut it open to find the sliver, but nothing appeared. It grew inflamed, the cutting I had made became a grand opening, the finger swelled to huge proportions, the hand did the same, and the fore arm did likewise. The skin came off the finger and I feared the loss of the finger, hand and arm. I was then living in the Turkish Baths at Newark, N. J. I had been treating it with cold water bandages, which relieved the pain, but contrary to all inflammatory difficulties only increased the trouble.

A gentleman came to the baths at this point, who seeing my trouble, said to me, "go to Robinson, at Bloomfield, who is well versed in felons." Arriving at Robinson's, I showed my hand. "Sweat it," said he. "If the game is to sweat it, doctor, I have the implements to do that beautifully at home, and I will take your advice.

On arriving at home, I prepared for a bath, went into the hot room at 140 degrees and remained there two hours, and the felon took a sweat *and so did I.* Next day, I went in one and a half hours, next day, same, the rest of several days following for one hour each, and from the time I took the first bath the felon began its retrograde, the arm reduced to its natural size, next the hand, then the finger; and the skin having pealed off the finger as the hand dried down, new skin appeared and in a week the pain was gone, the swelling gone, the hand nearly restored.

---

CURE FOR RHEUMATISM.—The genial gentleman and accomplished microscopist, E. H. Griffith, has sent us, from away off west somewhere, the "cure" for rheumatism given below. We spent a night together over the tube recently, and as we were complaining of a slight rheumatic attack then, he takes the first opportunity to send us a prescription. Here it is:

Sleep with your head toward the north. Wear a chest protector. Take nitrate of potash. Sleep with a big dog. Use magnetism. Put on mustard plasters, lemon juice, sage tea.—Wear sulphur in your shoes. Carry a piece of sulphur in your vest pocket. Try hard rubbing. Eat Wilson Packing Company's Compressed Cooked Corned Beef. Wear silk, white flannel, red flannel, buckskin. Make a necklace of the knots produced by the sting of an insect on Golden Rod, and wear it next the skin. Exercise and keep it off. Keep as quiet as possible. Take morphine, carbolic acid; soft soap bandaged with flannel will do. Do not eat eggs or potatoes. Eat anything you please. Do not smoke at all; smoke as much as you like. Take camphor. Drink nothing but beer. Do not drink anything but whisky. Drink no ardent spirits. Keep in the house. Take a ride out whenever you can get a free one. Carry a piece of alum in your pocket. Take Turkish baths. Bathe in hot water with pearl ash in it. Bathe in cold water frequently. Do not bathe at all until you are nearly well. Go to Arkansas Hot Springs. Go to Sharon Springs, to Saratoga, to Florida, to Bermuda, to the Sandwich Islands, to California, to the South of France, to Mexico, to South America. Wear a horse-chestnut in your left-hand breeches pocket. Wear a potato in the other. Take Constitution water. Don't eat onions; eat vegetables. Wrap joints with cotton, and cover with oiled silk. Get out on the prairies. High land is better for rheumatism. So is balm of life and magnetic salve. Rub with kerosene. Put mustard poultice over the heart. Put on slippery-elm poultice. Go to Bodines and camp out, in the rain. If you don't effect a cure by doing as above, try something else.

We haven't tried it yet; when we do, will get E. H. G. to sit up with us and have him take a portion of the medicine himself.

